# Observation of Electric-Field-Induced Structural Dislocations in a Ferroelectric Oxide

**DOI:** 10.1021/acs.nanolett.0c04816

**Published:** 2021-04-16

**Authors:** Donald M. Evans, Didrik René Småbråten, Theodor S. Holstad, Per Erik Vullum, Aleksander B. Mosberg, Zewu Yan, Edith Bourret, Antonius T. J. van Helvoort, Sverre M. Selbach, Dennis Meier

**Affiliations:** † Department of Materials Science and Engineering, Norwegian University of Science and Technology (NTNU), 7491 Trondheim, Norway; ‡ SINTEF Industry, 7491 Trondheim, Norway; § Department of Physics, Norwegian University of Science and Technology (NTNU), 7491 Trondheim, Norway; ∥ Department of Physics, ETH Zürich, 8093 Zürich, Switzerland; ⊥ Materials Sciences Division, Lawrence Berkeley National Laboratory, Berkeley, California 94720, United States

**Keywords:** Nanotechnology, ferroelectric, semiconductors, functional oxide, dislocations, hexagonal manganites

## Abstract

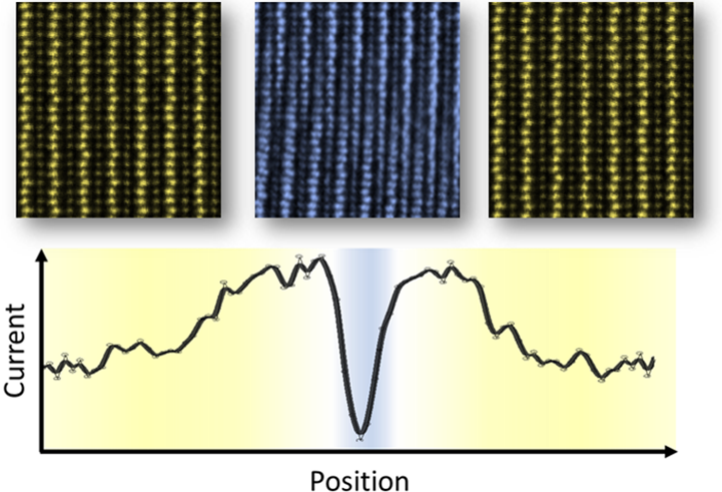

Dislocations are 1D topological defects with emergent electronic properties. Their low dimensionality and unique properties make them excellent candidates for innovative device concepts, ranging from dislocation-based neuromorphic memory to light emission from diodes. To date, dislocations are created in materials during synthesis via strain fields or flash sintering or retrospectively via deformation, for example, (nano)-indentation, limiting the technological possibilities. In this work, we demonstrate the creation of dislocations in the ferroelectric semiconductor Er(Mn,Ti)O_3_ with nanoscale spatial precision using electric fields. By combining high-resolution imaging techniques and density functional theory calculations, direct images of the dislocations are collected, and their impact on the local electric transport behavior is studied. Our approach enables local property control via dislocations without the need for external macroscopic strain fields, expanding the application opportunities into the realm of electric-field-driven phenomena.

## Introduction

The presence of dislocations transcends condensed matter research and gives rise to a diverse range of emergent phenomena,^1−6^ ranging from geological effects^7^ to light emission from diodes.^8^ Classically, dislocations have often been regarded as imperfections in otherwise perfectly ordered crystals that are detrimental to electronic functionality.^9,10^ However, very recently, there has been growing interest in the properties of individual dislocations *because* they have different symmetries, and therefore different properties, from those of the surrounding material.^11^ Examples of emergent functional physical properties include locally enhanced conductivity,^12^ ferromagnetic order in antiferromagnets,^13^ redox-based resistive switching behavior,^4^ and trapping of light.^14^ Because of the low dimensionality of dislocations, which makes them highly stackable, and their unique properties, dislocations are now perceived as promising atomic-scale entities for next-generation device applications.^10,11^ Despite their exciting application potential, there are key limitations to the technological development of dislocations: The most critical is the control of their formation. Currently, dislocations are created by strain engineering, predominantly in one of two ways: either strain fields during growth, so that dislocations form to release the strain,^15−17^ or postgrowth via applied stress,^18^ for example, by nanoindentation.^19−22^ Although strain engineering is a highly efficient tool for creating dislocations, it is challenging to alter the properties of a material exactly via strain fields on the local scale. This is particularly problematic after a material has been implemented into a device architecture. Aside from strain, flash sintering has been applied to produce defects, including structural dislocations. This approach is particularly promising regarding the production of defect-rich samples, but it lacks the nanoscale control required for technological applications.^23^

Here we use electric fields to create partial dislocations in a ferroelectric material with nanoscale spatial precision, altering the structure and the electronic transport behavior where the field was applied. The hexagonal manganite Er(Mn,Ti)O_3_ is chosen as the model system (see the Materials and Methods for details),^24,25^ but similar behavior is expected in structurally equivalent systems such as hexagonal gallates, indates, and ferrites.

## Results

### Electric-Field Creation of Structural Defects

**Figure 1:**
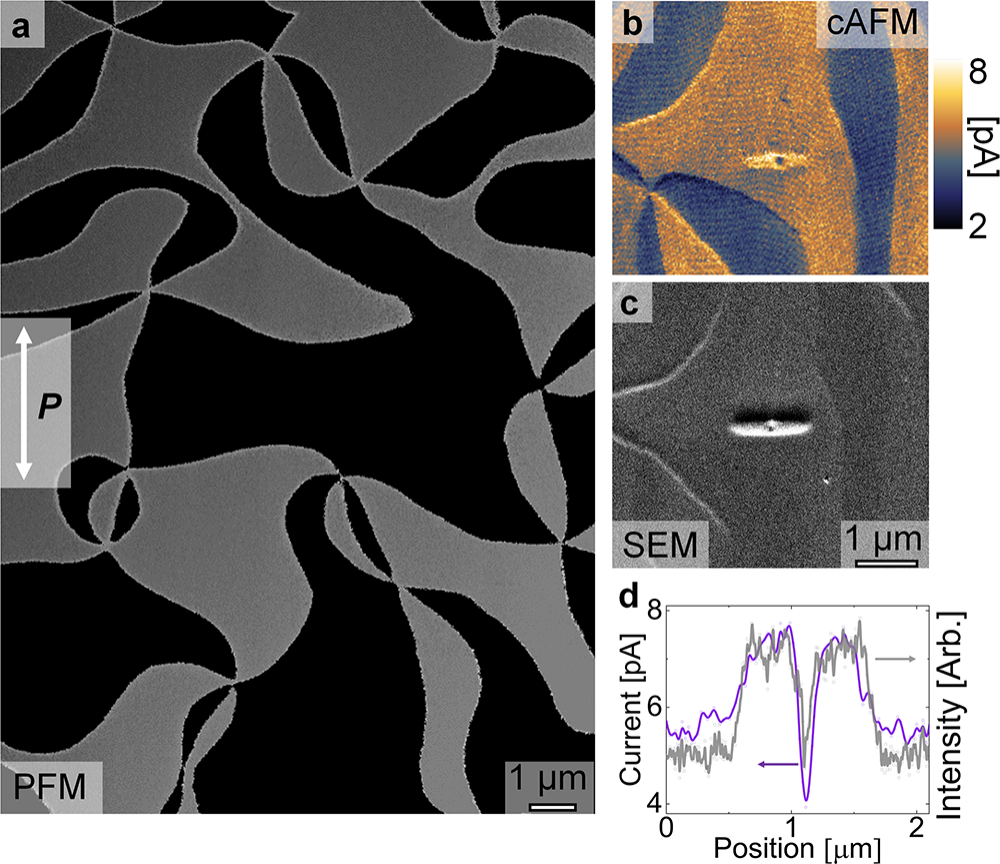
Electric-field control of local conductance in Er(Mn,Ti)O_3_. (a) Lateral dual AC resonance tracking (DART) mode PFM phase image of an in-plane ferroelectric domain pattern, taken with an amplitude of 5V and a frequency range of 784.5–794.5 kHz. Bright and dark contrasts represent ferroelectric 180° domains, revealing the characteristic domain pattern of hexagonal manganites. (b) cAFM scan recorded with +45 V on the same sample after the application of −60 V for 5 s to the back electrode with a stationary AFM tip; the position where the tip was placed coincides with the blue center of the gold elliptical feature seen in the cAFM map. Bright (gold) colors correspond to areas of higher conductance, and dark (blue) colors correspond to areas of lower conductance. (c) SEM image of the feature in panel b demonstrating conductivity-sensitive SEM contrast. (d) Cross-sectional graph, correlating SEM contrast (grey) with cAFM contrast (purple). The images in panels b and c represent a larger view of the same area discussed in ref (30).

The ferroelectric domain structure of (110)-oriented Er(Mn,Ti)O_3_ is shown in the piezoresponse force microscopy (PFM) image in Figure 1a. The PFM data demonstrate that the two possible 180° polarization domains, +*P* and −*P*, form six-fold meeting points that are characteristic of hexagonal manganites.^26,27^ After mapping the domain structure of Er(Mn,Ti)O_3_, we apply −60 V (for 5 s) to the back electrode while keeping the probe tip static. Using a simple point-charge approximation (*E* = *V*/*r*_tip_), the biased static tip generates an electric field of ∼6 MV/cm.^28,29^ Subsequent conductive atomic force microscopy (cAFM) images (taken with +45 V) reveal a local modification of the transport behavior, manifesting as an ellipsoidal area of enhanced conductance (gold, in Figure 1b) with an insulating core: The core of reduced conductance occurs directly below the position of the AFM tip, that is, the position of the maximum electric field. A scanning electron microscopy (SEM) image of the same region as in Figure 1b is presented in Figure 1c, which demonstrates that the area of enhanced conductance in the cAFM scan correlates with bright contrast in SEM, associated with an increased yield of secondary electrons. A direct comparison of the SEM and cAFM images is given by the cross-sectional profiles in Figure 1d, showing a clear correlation. Figure 1 thus reveals that the electrically modified region displays two regimes: (i) a broader area with enhanced conductance due to anti-Frenkel defects, as reported in ref (30), and (ii) a highly localized area with reduced conductance. In contrast with the enhanced conductance in regime (i), the suppressed conductance in regime (ii) cannot be explained by the same defect formation process,^30^ indicating a different microscopic origin.

### Atomic-Scale Imaging of Defects

**Figure 2:**
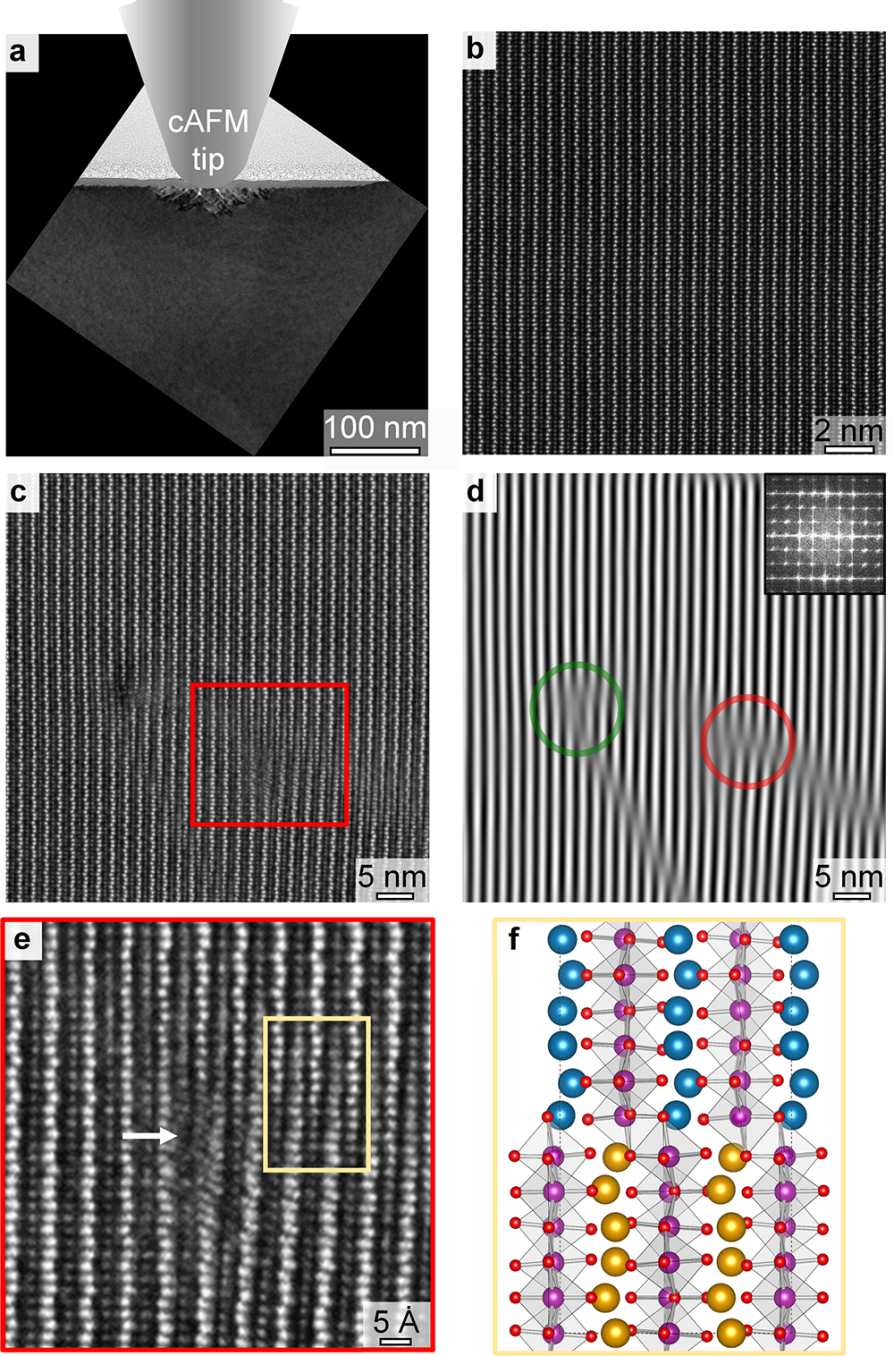
Atomic-scale structure of electrically induced dislocations and stacking faults. (a) Bright-field TEM image and schematic of the cAFM tip, illustrating that the perturbed crystal structure is restricted to the area beneath the cAFM tip. (b) HAADF-STEM image viewed down the [1̅00] direction, showing the as-grown state. (c) HAADF-STEM image viewed down the [1̅00] direction, showing the region beneath the position of the AFM tip after the application of the electric field. The periodic crystal structure is interrupted by line features extending in directions close to the ⟨011⟩ directions. The bright and gray dots are Er and Mn atomic columns, respectively. (d) Inverse fast Fourier transformation (FFT) obtained by selecting only the (002) maxima of an FFT of panel b; see the insert. The image shows areas with the same periodicity of the Er lattice, allowing easier identification of lattice defects. Two edge dislocations are highlighted by red and green circles. (e) Representative HAADF-STEM image taken across the crystallographic features, corresponding to the area marked by the red box in panel c. (f) Fully relaxed DFT supercell, modeling the dislocated structure by unit cells dislocated by *c*/4 and *a*/3 with respect to each other. Large gold and blue spheres represent Er atoms on either side of the stacking fault; Mn and O atoms are sketched in purple and red, respectively.

To investigate the underlying microscopic effect, we study the atomic structure in the electrically modified region using transmission electron microscopy (TEM) techniques on a focused ion beam (FIB) prepared lamella. (See the Materials and Methods.) A bright-field overview image of a lamella, prepared to cross-section the electrically modified region in Figure 1, is given in Figure 2a. The data show that the crystal structure has changed in a limited region around the position where the cAFM tip was placed (indicated schematically). This modified region has a lateral extension of ∼160 nm and protrudes ∼35 nm into the depth of the sample, whereas the rest of the crystal structure has remained in the as-grown state. To discern the differences between the modified region and the as-grown state,^26,30,31^ high-angle annular dark-field scanning transmission electron microscopy (HAADF-STEM) images of the two regions are presented in Figure 2b,c, respectively. In Figure 2b,c, the bright dots correspond to Er and Mn atomic columns; the heavier Er atoms give brighter dots than the lighter Mn atoms. The HAADF-STEM of Figure 2c shows that the Er layers are disrupted in several places and appear to be displaced, seemingly merging with the Mn layers in the projected image. This disruption of the lattice only occurs directly underneath the cAFM tip, and no such alterations of the lattice are seen in the wider crystal (Figure 2a). To visualize associated local changes in the lattice periodicity, Fourier filtering is applied, as presented in Figure 2d. This treatment reveals discontinuities in the lattice periodicity and the presence of edge dislocations, marked by the green and red circles in Figure 2d. An enlarged section with a higher resolution of the feature in the red box in Figure 2c is shown in Figure 2e. On the left side of the dislocation in Figure 2e, the HAADF-STEM image shows the normal sequence of alternating Er and Mn layers, whereas the right side of the image shows Er columns that continuously merge into Mn columns and vice versa. We note that there is no clear interface where Er columns swap with Mn columns, as the interface is inclined to the [001] viewing direction. The general possibility to stabilize such stacking faults is not surprising,^32^ as their growth-induced formation has been reported in hexagonal manganites and ferrites.^33−35^ In contrast with previous work, however, the stacking faults in our system are generated postgrowth and with nanoscale spatial precision by the application of an electric field. We note that the sample used in this work has been cut from the same single crystal studied in, for example, refs (24 and 30) and discussed in, for example, refs (36 and 37). During the previous extensive high-resolution TEM investigations, no partial dislocations were observed in the ErMnO_3_ crystal lattice, corroborating that such defects are not intrinsic to the lattice or generated by the applied preparation methods.

To evaluate the stability of the defect structure resolved in Figure 2e, we model the dislocated lattice using density functional theory (DFT), as illustrated in Figure 2f. To look for stable states, we consider the isostructural system YMnO_3_ (refs (31 and 38)), where multiple 240-atom 1 × 8 × 1 supercells were initialized in the high-symmetry *P*6_3_/*mmc* phase with different dislocation configurations and relaxed into the polar state *P*6_3_*cm* until the forces on all of the atoms were <0.01 eV Å^–1^. The analysis of YMnO_3_ instead of ErMnO_3_ is standard practice, as their structural and chemical properties are very similar (see Table S1 and Figure S1), but the absence of f electrons significantly reduces the computational cost.^31,38,39^ The DFT calculations show that a stable dislocated structure exists when the lattice is displaced by *c*/4 and *a*/3 (or one of the six symmetry equivalent directions, perpendicular to the unique axis), as shown in Figure 2f. The calculated dislocation configuration is in excellent agreement with our HAADF-STEM data (Figure S2 shows the dislocated structure viewed down the [110] direction, which is symmetry equivalent to [1̅00]), leading us to the conclusion that the merging features in Figure 2e correspond to a stacking fault between two partial dislocations.

**Figure 3:**
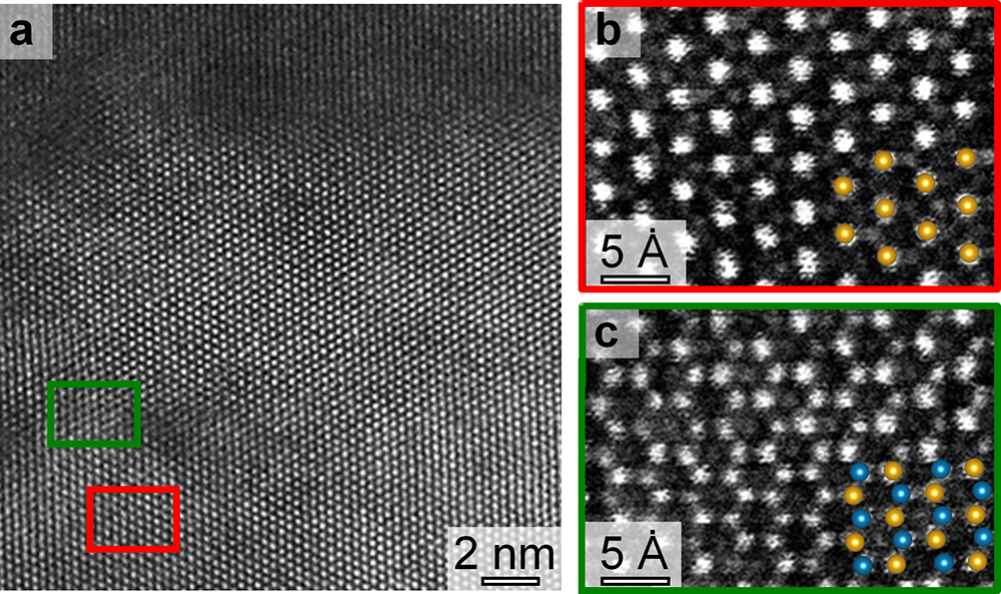
Atomic defect structure viewed along the [001] axis. (a) HAADF-STEM image of a region with electric-field induced defects, written with the same parameters as used in Figures 1 and 2. Bright dots correspond to Er atom columns. (b) HAADF-STEM image of the area represented by the red box of panel a, showing the expected Er cation lattice for a hexagonal crystal when viewed along the [001] direction. The gold dots correspond to a DFT simulation of the structure, looking down the [001] axis. Mn columns can be seen as weak dots in between the more pronounced dots from the Er columns. (c) HAADF-STEM image corresponding to the area represented by the green box in panel a, showing the Er pattern that deviates from the unperturbed crystallographic structure seen in panel b. Gold and blue dots represent the bulk and shifted Er atom columns when the stable dislocation of Figure 2 is viewed down the [001] axis. The overlay shows that the dislocated structure reproduces the pattern resolved by HAADF-STEM.

On the basis of the DFT calculations, we can predict the expected atomic pattern when we image along other crystallographic directions, allowing us to conduct an independent test experiment. The calculated crystal structure viewed along different directions, with and without the dislocated structure, is presented in Figure S3, including the superposition of the dislocated structure and the unperturbed Er(Mn,Ti)O_3_ structure with space group symmetry *P*6_3_*cm.*^40^ This superposition accounts for the depth convolution present within TEM lamellas with finite thickness. A representative HAADF-STEM image recorded along the [001] direction of an Er(Mn,Ti)O_3_ lamella with electrically modified regions (analogous to Figure 1) is presented in Figure 3a. As in Figure 2, the modification of the lattice only occurs in the area exposed to the high electric field. The HAADF-STEM image shows the Er atoms as bright dots, reflecting the characteristic pattern associated with the *P*6_3_*cm* space group symmetry of the crystal sketched in Figure S3. The latter is confirmed by the high-resolution HAADF-STEM image in Figure 3b, taken from the area represented in red in Figure 3a. On closer inspection, however, local deviations from the ideal structure are observed, as shown by the HAADF-STEM image in Figure 3c (obtained in the area represented in green in Figure 3a). Here the Er atoms are found to form a close-packed honeycomb-like structure, as illustrated by the schematic overlay, in accordance with the DFT-predicted structure. (See Figure S3; blue and gold dots in the overlay represent Er atoms from volumes on either side of the stacking fault.)

### Calculated Defect Properties

**Figure 4:**
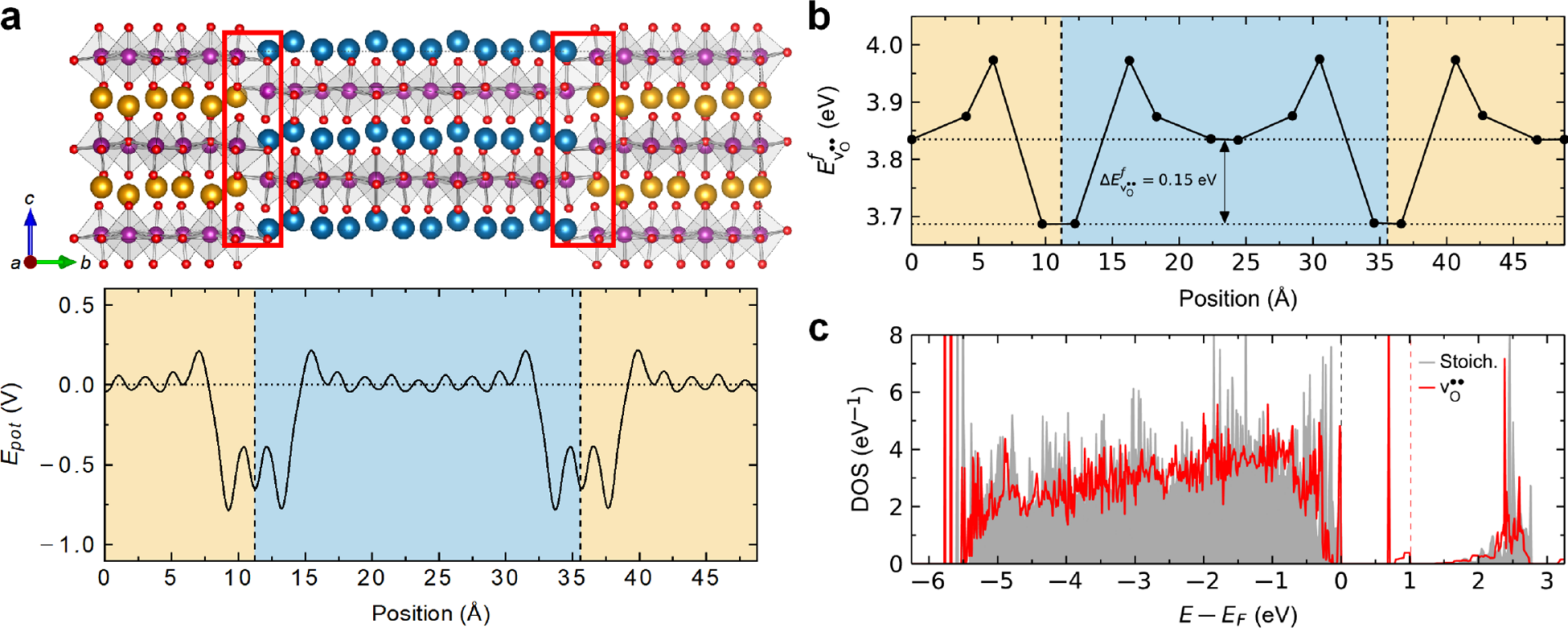
Calculation of the electronic defect structure. (a) Dislocated structure (upper part) and electrostatic potential (lower part) calculated by density functional theory (DFT). The calculated electrostatic potential is scaled relative to the potential away from the dislocated interfaces, which are indicated by the red boxes. The significant reduction in the potential (ca. 1/2*E*_g_ at the interface) corresponds to bound negative charges, which are likely to attract positively charged **v**_**O**_^••^. (b) **v**_**O**_^••^ formation energy with increasing distance from the interface(s), showing a significant energy preference, 0.15 eV, for forming **v**_**O**_^••^ at the interface(s) compared with the bulk. (c) Calculated local DOS at the partial dislocations in a stoichiometric supercell (gray) and with an accumulation of oxygen vacancies (red). The corresponding Fermi levels for the pristine cell and the **v**_**O**_^••^ defect cell are marked by dashed gray and red lines in panel c, respectively. The DOSs are aligned to core states.

After establishing the presence of the structural defects in the region of reduced conductance, we calculate the associated formation energy. Note that because of the thermal stability of the structural ground state and the ferroelectric polarization,^41−43^ finite temperature effects would be subtle at room temperature and would not change the results qualitatively, and hence they are not considered. For the partially dislocated structure in Figure 2f, we find a formation energy of ∼755 mJ m^–2^, which is only about seven times higher than for the charged domain walls, which naturally occur in the hexagonal manganites.^44^ The DFT calculations further show that the dislocated structure significantly alters the electrostatic potential. To quantify this, we calculate the planar average across the supercell in Figure 4a, which shows a decrease in the potential energy of ∼0.75 eV (∼1/2*E*_g_) compared with the bulk, corresponding to bound negative charges associated with the stacking fault. However, in contrast with the negatively charged tail-to-tail domain walls in hexagonal manganites,^44^ neither calculated Bader charges nor Mn magnetic moments reveal any inherent electronic charge transfer between the dislocated structure and the bulk. This is because the magnitude of the band bending (visualized in Figure S4) does not fulfill the Zener-like breakdown criterion,^44^ meaning that our defects are not compensated by electronic charge carriers.

However, the bound negative charges associated with the dislocated structure in Figure 4a can attract mobile ionic defects with a relative positive charge, such as oxygen vacancies (**v**_**O**_^••^). In fact, we find that the **v**_**O**_^••^ formation energy is reduced by ∼0.15 eV compared with the bulk (Figure 4b), demonstrating a significant defect segregation enthalpy. To contextualize the strength of this driving force, it is five times higher than the attraction of oxygen interstitials (**O**_**i**_^″^) to neutral domain walls in the hexagonal manganites (∼0.03 eV), which are experimentally well documented to have enhanced conductance.^45^ As such, the five times higher driving force is expected to pin **v**_**O**_^••^ to the stacking faults and vice versa. To illustrate the corresponding changes in the electronic state, we calculate the density of states (DOS) in YMnO_3_ with and without **v**_**O**_^••^ at the dislocated structure (Figure 4c). The DOSs are qualitatively similar, but the **v**_**O**_^••^ DOS has a strongly localized state within the band gap and a higher Fermi energy. This increase in Fermi energy at the interface gives a variation in electronic charge carrier concentration and—in a p-type system with holes as the majority carriers—^38^ is expected to cause a diffusion current, leading to a local reduction in the number of available electronic charge carriers. As a consequence, the dislocation-induced structural changes enhance the resistivity relative to the bulk, which is consistent with the lower conductance observed in the cAFM data in Figure 1b.

## Discussion

Our experimental observations and DFT results reveal two important criteria for the formation of dislocations in ferroelectric oxides under locally applied voltage, promoting their injection postgrowth and with nanoscale spatial precision: (i) The cation has access to a new (meta-)stable cation configuration and (ii) the applied electric field generates enough energy to overcome the barrier toward the new (meta-)stable state without leading to amorphization from leakage currents. A possible driving force for the spontaneous dislocation of the lattice observed in Er(Mn,Ti)O_3_ is electric-field gradients that act on the uniaxial polarization (*F* = *P*·∇*E*). At present, however, no microscopic model exists that can capture the emergence of dislocations in electric fields, and theoretical in-depth studies are highly desirable.

## Conclusions

Our work demonstrates that electric fields can be used to create partial dislocations in a complex oxide to engineer the local structure and electronic response, bypassing the necessity of applied strain fields. The ability to induce such changes postgrowth, on demand, and with nanometer spatial precision provides a conceptually different approach to local property engineering, as exemplified by the enhanced resistance in hexagonal manganites. Our approach is expected to be generally applicable to dielectric materials close to a structural instability, where a sufficiently large electric field can build up. Because the electric-field-driven dislocation injection is applicable after a material has been synthesized or integrated into a device, it presents an opportunity for engineering functional materials via low-dimensional structural defects.

## Materials and Methods

### Samples

Single crystals of Er(Mn_1–*x*_,Ti_*x*_)O_3+δ_ with *x* = 0.002 were grown using the pressurized floating zone method (ref (25)). From this, we prepared samples with the ferroelectric polarization parallel to the sample surface (in-plane *P*). The crystals were orientated by Laue diffraction, cut to have thicknesses of ∼0.5 mm, and electromechanically polished to give a root-mean-square (RMS) surface roughness of ∼1 nm.

### Focused Ion Beam

TEM specimen preparation was carried out using a Thermo Fisher Scientific Helios G4 UX DualBeam FIB. Lamellas were prepared “flipped” with *in situ* lift-out and backside milling. Final polishing was carried out at 2 kV. High-resolution Pt markers were used in conjunction with C protection layers to ensure that the final lamellas were centered on the region with suppressed conductance.

### Transmission Electron Microscopy

TEM analysis was performed using a double-Cs aberration-corrected cold field emission gun JEOL ARM 200FC apparatus operated at 200 kV. STEM imaging was performed with a 27 mrad semiconvergence angle.

### Atomic Force Microscopy

The lateral DART–PFM was performed on a Cypher ES Environmental AFM using an Oxford Instruments Asylec-01-R2 Pt/Ir tip. The remaining scanning probe microscopy (SPM) measurements were performed on an NT-MDT NTEGRA SPM using a TipsNano DCP20 tip. The voltage was applied to the sample back-electrode with the tip connected to ground.

### Density Functional Theory

Calculations on isostructural YMnO_3_ (isostructural and electronically similar to ErMnO_3_ but without complicating f electrons) were carried out using the projector augmented wave (PAW) method, as implemented in the Vienna Ab initio Simulation Package (VASP),^46−48^ using the Y_sv Mn_sv and standard O pseudopotentials supplied with VASP. PBEsol+U^49,50^ with *U* = 5 eV on Mn 3d states, combined with a collinear frustrated antiferromagnetic order on the Mn sublattice, was used to reproduce the experimental lattice parameters^43^ and the electronic band gap.^51^ The energy cutoff of the plane-waves was set to 550 eV. The Brillouin zone was sampled with a Γ-centered 4 × 1 × 2 *k*-point grid for geometry optimization and a 6 × 1 × 3 grid for DOS and electrostatic potential calculations. 240-atom 1 × 8 × 1 supercells were initialized in the high-symmetry *P*6_3_/*mmc* phase with different dislocation configurations and relaxed until the forces on all atoms were <0.01 eV Å^–1^. Ferroelectric polarizations were calculated from a simple point-charge model using formal charges.^52^ The dislocation formation energy was calculated as
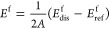
where *E*_di*s*_^f^ and *E*_ref_^f^ are the energies of a dislocation supercell and a monodomain supercell, respectively, and *A* is the cross-sectional area.

## Supporting Information Available

Comparison of the calculated electronic properties of YMnO_3_ and ErMnO_3_, DFT simulations of how the dislocations would look in different viewing directions and the depth effects, and calculations of the changes in electronic orbitals at the dislocations. (PDF)
