# Supplementary material for: Observation of Electric-Field-Induced Structural Dislocations in a Ferroelectric Oxide
Source: Nano Lett. 2021 Apr 16;21(8):3386–92. doi: 10.1021/acs.nanolett.0c04816 (PMC8155316; doi:10.1021/acs.nanolett.0c04816)
Supplement: Supplementary file 1 — nl0c04816_si_001.pdf [file nl0c04816_si_001.pdf]

Observation of Electric-Field-Induced Structural Dislocations in a Ferroelectric Oxide

Donald M. Evans,[1†] Didrik René Småbråten,[1‡] Theodor S. Holstad,[1] Per Erik Vullum,[2] Aleksander B. Mosberg,[3] Zewu Yan,[4,5] Edith Bourret,[5] Antonius T. J. van Helvoort,[3] Sverre M. Selbach,[1] and Dennis Meier[1*]*

**E-mail: donald.evans@uni-a.de and dennis.meier@ntnu.no*

[1] Department of Materials Science and Engineering, Norwegian University of Science and Technology (NTNU), Trondheim, Norway.

[2] SINTEF Industry, 7491 Trondheim, Norway.

[3] Department of Physics, Norwegian University of Science and Technology (NTNU), Trondheim, Norway.

[4] Department of Physics, ETH Zürich, 8093 Zürich, Switzerland.

[5] Materials Sciences Division, Lawrence Berkeley National Laboratory, Berkeley, California 94720, USA.

Supplementary Materials

Table S1. Comparison of calculated *a* and *c* lattice parameters, bulk polarization (*P*), apical (θ) and planar (α) MnO_5 tilt angles, and electronic band gap (E_g) in $YMnO_3$ and $ErMnO_3$.

Parameter	$YMnO_3$	$ErMnO_3$
a (Å)	6.100	6.049
c (Å)	11.420	11.370
P ($\mu C/cm^2$)	7.051	7.968
θ (°)	5.107	5.273
α (°)	7.082	7.569
E_g (eV)	1.531	1.565

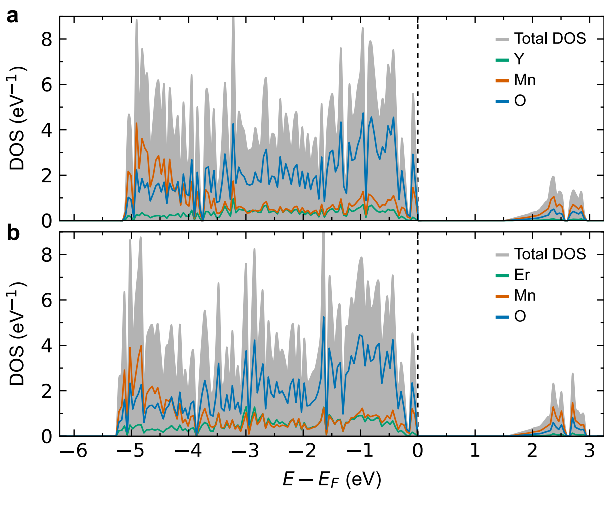

Figure S1. Calculated electronic density of states (DOS) of **a** $YMnO_3$, and **b** $ErMnO_3$.

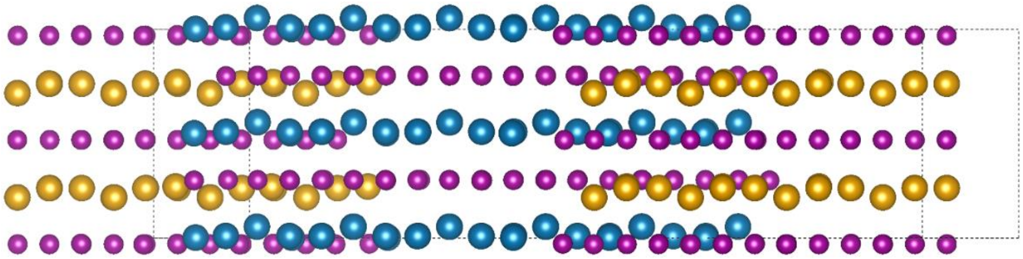

Figure S2 | A *c*/4 and *a*/3 dislocated structure viewed at 60 º to the interface. DFT calculations showing one of the symmetrically equivalent ***c***/4, ***a***/3, dislocated structures viewed along the [110] direction, or symmetrically equivalent projections. In this case, there is a continual merging of the *R*-cation and Mn atomic columns, as observed in the HAADF-STEM of Figure 2**c**.

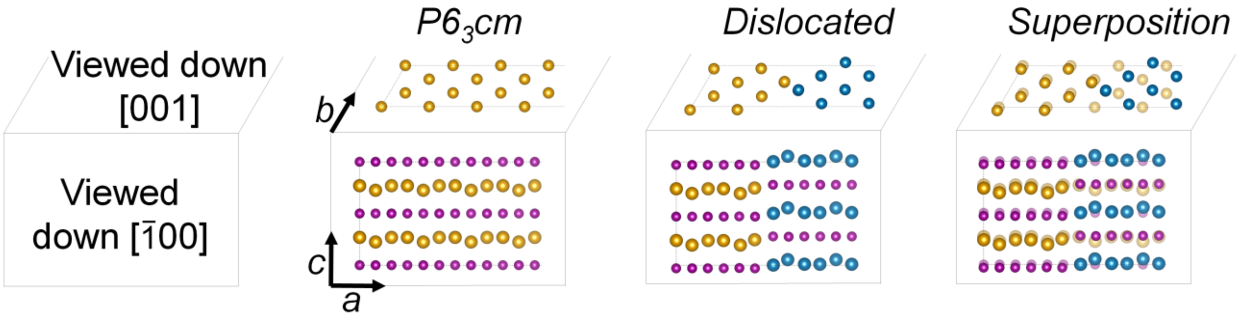

Figure S3 | Viewing directions and depth effects. Left: Illustration of the atomic structure in the unit cell of hexagonal manganites with space group symmetry $P6_3cm$, viewed along the [001] and [$\bar{1}$00] directions. Er and Mn atoms are represented by gold and purple spheres, respectively. Middle: Comparison of dislocated structures; Er (gold and blue) and Mn (purple). Right: Superposition of the structures seen on the left and in the middle.

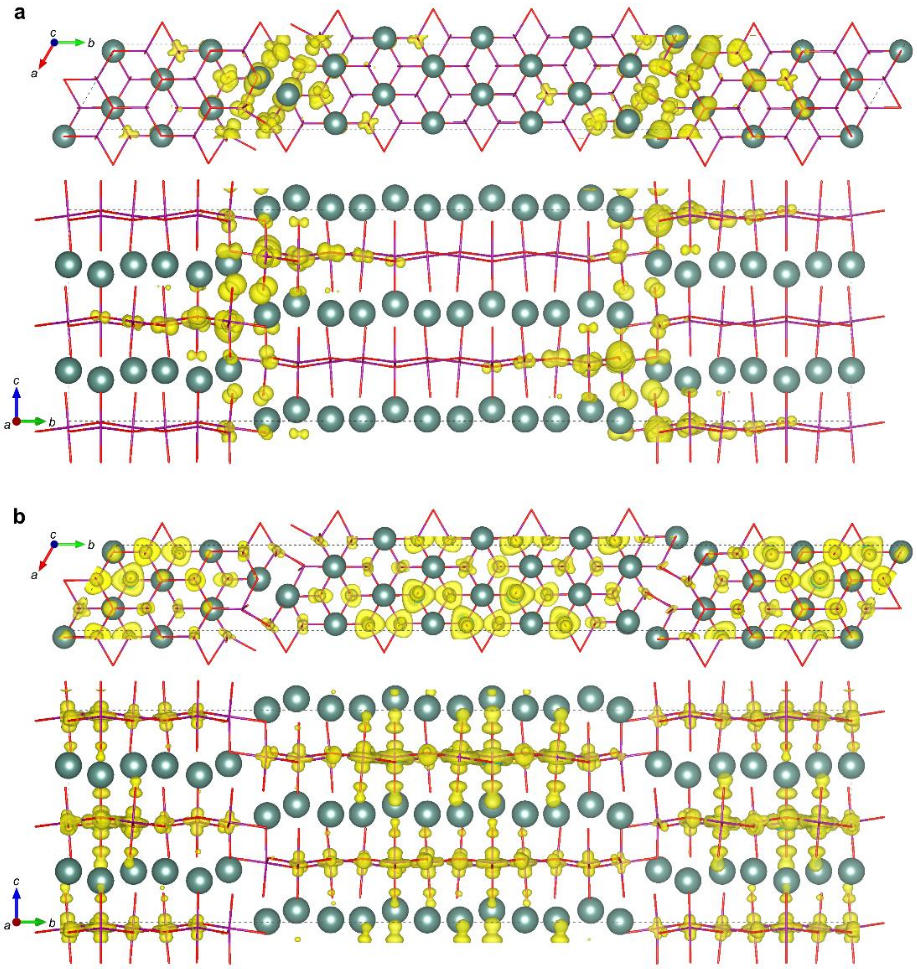

Figure S4 | Change in local orbitals at a dislocation. a, Visualization of the calculated partial charge density for the highest occupied wavefunction, illustrating the position of the valence band edge in the supercell. **b**, Visualization of the calculated partial charge density of the lowest unoccupied wavefunction, illustrating the position of the conduction band edge in the supercell.
